# Strain-stress study of Al_*x*_Ga_1−*x*_N/AlN heterostructures on c-plane sapphire and related optical properties

**DOI:** 10.1038/s41598-019-46628-4

**Published:** 2019-07-15

**Authors:** Yining Feng, Vishal Saravade, Ting-Fung Chung, Yongqi Dong, Hua Zhou, Bahadir Kucukgok, Ian T. Ferguson, Na Lu

**Affiliations:** 1 Lyles School of Civil Engineering, Birck Nanotechnology Center, Purdue University, West Lafayette, IN 47907 USA; 2 Applied Materials Division, Argonne National Laboratory, 9700 South Cass Ave, Lemont, IL 60439 USA; 3 Electrical and Computer Engineering, Missouri University of Science and Technology, Rolla, MO 65409 USA; 4 National Synchrotron Radiation Laboratory, University of Science and Technology of China, Hefei, Anhui 230026 China; 5 Advanced Photon Source, Argonne National Laboratory, 9700 South Cass Ave, Lemont, IL 60439 USA; 6 Southern Polytechnic College of Engineering and Engineering Technology, Kennesaw State University, Marietta, GA 30060 USA; 7 School of Materials Engineering, Purdue University, West Lafayette, IN 47907 USA

**Keywords:** Semiconductors, Materials science

## Abstract

This work presents a systematic study of stress and strain of Al_*x*_Ga_1−*x*_N/AlN with composition ranging from GaN to AlN, grown on a *c*-plane sapphire by metal-organic chemical vapor deposition, using synchrotron radiation high-resolution X-ray diffraction and reciprocal space mapping. The *c*-plane of the Al_*x*_Ga_1−*x*_N epitaxial layers exhibits compressive strain, while the *a*-plane exhibits tensile strain. The biaxial stress and strain are found to increase with increasing Al composition, although the lattice mismatch between the Al_*x*_Ga_1−*x*_N and the buffer layer AlN gets smaller. A reduction in the lateral coherence lengths and an increase in the edge and screw dislocations are seen as the Al_*x*_Ga_1−*x*_N composition is varied from GaN to AlN, exhibiting a clear dependence of the crystal properties of Al_*x*_Ga_1−*x*_N on the Al content. The bandgap of the epitaxial layers is slightly lower than predicted value due to a larger tensile strain effect on the *a*-axis compared to the compressive strain on the *c*-axis. Raman characteristics of the Al_*x*_Ga_1−*x*_N samples exhibit a shift in the phonon peaks with the Al composition. The effect of strain on the optical phonon energies of the epitaxial layers is also discussed.

## Introduction

III-Nitride alloys have attracted considerable attention in a wide range of applications of optical, optoelectronic, high-power, and high-frequency devices such as light emitting diodes (LEDs), laser diodes, and high electron mobility transistors (HEMTs)^1–6^. For instance, the hexagonal Al_*x*_Ga_1−*x*_N is one of the most promising candidates for ultraviolet (UV)-LED applications, especially because of its wide bandgap (E_g_) range from 3.42 eV (for GaN) to 6.2 eV (for AlN) at room temperature^7^. Al_*x*_Ga_1−*x*_N is also an optimum intermediate layer for InGaN-based LEDs and InAlN transistors^2,8–10^. Al_*x*_Ga_1−*x*_N/GaN HEMTs paves the way for achieving high power radio frequency (RF) devices due to high electron mobility, large critical breakdown field, high sheet charge density, high electron saturation velocity, and high temperature operation^11^. Al_*x*_Ga_1−*x*_N/AlN heterostructure combines the photodetector abilities of deep ultra-violet (DUV) AlN along with the tunable bandgap Al_*x*_Ga_1−*x*_N, thereby also suppressing the visible spectrum and enhancing the UV/visible rejection ratio^12,13^. This has applications in military target or missile detection, biochemical sensing, as solar-blind detectors, air/water purification, curing, and biomedical therapies and instrumentation^14–17^. Considering the photodetector applications, AlN has a higher bandgap than Al_*x*_Ga_1−*x*_N and hence the resulting photodetector spectrum (of light waves incident on Al_*x*_Ga_1−*x*_N surface) would be dominantly dependent on the Al_*x*_Ga_1−*x*_N epitaxial layer properties; as opposed to other structures consisting of Al_*x*_Ga_1−*x*_N and a lower bandgap material, where it could be difficult to separate the effects of the two materials on the energy spectrum. Also, an Al_*x*_Ga_1−*x*_N/AlN structure would have the flexibility to function as a photodetector from top and bottom sides with front and back illumination respectively, with the top Al_*x*_Ga_1−*x*_N epitaxial layer having bandgap range from ~4 eV to ~6 eV depending on the Al content, and a bottom AlN layer with 6.2 eV band gap. Using an AlN intermediate layer for Al_*x*_Ga_1−*x*_N could also improve the crystal quality of the heterostructure and reduce absorption losses^16^. However, highly efficient and reliable electronic and optoelectronic devices require epitaxial layers with excellent crystal quality (i.e., low dislocation density and residual strain). It is challenging to grow high-quality Al_*x*_Ga_1−*x*_N thin films, particularly with high Al composition (*x*); this is due to the lattice mismatch and thermal expansion difference between the thin films and substrates, which generally results in high-level strain-stress and mosaicity^18–20^. Strain-stress in epitaxial layers is one of the leading factors that reduces the electron mobility and degrades the device performance^21–23^. Also, their optical and morphological properties could be improved by reducing the strain and stress. Therefore, it is vital to understand the strain and stress mechanism for improving the optical and electronic properties and applications of III-Nitrides.

High-resolution X-ray diffraction (HRXRD) and reciprocal space mapping (RSM) could be used to understand the crystal properties and to analyze the strain and stress in epitaxially grown III-Nitride films^24^. The effect of different intermediate layers such as AlN, GaN, and step-graded Al_*x*_Ga_1−*x*_N for Al_*x*_Ga_1−*x*_N/GaN HEMT structures on silicon (111) substrate has been studied by XRD, RSM and Hall effect measurements, showing that the in-plane stress can largely affect the two-dimensional electron gas mobility and carrier concentration^25^.

The origin of stresses in Al_*x*_Ga_1−*x*_N/GaN heterostructures grown on *c*-plane sapphire substrate relies mainly on the thickness and growth temperature of the layers, alloy composition, device structure, and doping^20,23,26–28^. In the case of Al_0.4_Ga_0.6_N/AlN/GaN(superlattices)/GaN/sapphire and Al_0.6_Ga_0.4_N/AlN/sapphire, stress was released due to misfit dislocations at several interfaces in the heterostructure due to composition pulling effect^29^. Also, strain and threading dislocations accumulation increased at step edges in Λ-shape distributed Al_x_Ga_1−x_N (*x* from 7% to 30%) grown on AlN/GaN/sapphire substrates^30^. In the case of a GaN/Al_x_Ga_1−x_N (graded *x* from 0 to 26 and 42%)/GaN/sapphire structure, a tensile strain was observed in the Al_*x*_Ga_1−*x*_N and a compressive strain in the GaN cap layer; also, crystal coherence was broken at the interfaces but it was consistent within the Al_*x*_Ga_1−*x*_N layers^31^. Crystal defects and dislocations could be attenuated by growing a high temperature (HT) AlN intermediate layer as reported in the case of Al_*x*_Ga_1−*x*_N/AlN (HT)/GaN/sapphire^32^ and by modifying or reducing the interfaces.

However, a systematic study of strain and stress in Al_x_Ga_1−x_N/AlN heterostructures, especially for high *x* (>0.5) Al_*x*_Ga_1−*x*_N epitaxial layers, on *c*-plane sapphire substrates by synchrotron radiation HRXRD and RSM technique has not been reported. It is crucial to study the crystal properties of Al_*x*_Ga_1−*x*_N/AlN structures, which is a step towards improving their quality and potential for practical applications.

In this work, the overall strain, biaxial strain, hydrostatic strain, and biaxial stress along the *a*- and *c*-axis, are analyzed and calculated for Al_*x*_Ga_1−*x*_N/AlN heterostructure on sapphire substrates with varying *x* and Al_*x*_Ga_1−*x*_N composition from GaN to AlN using synchrotron radiation HRXRD and RSM. The epitaxial layers have a good surface quality and are free of cracks. The effect of the Al content on the crystal properties, dislocation densities and coherence lengths are discussed. The effect of strain on the optical properties of the Al_*x*_Ga_1−*x*_N thin films has been investigated using photoluminescence (PL) and Raman spectroscopy.

## Results and Discussion

**Figure 1:**
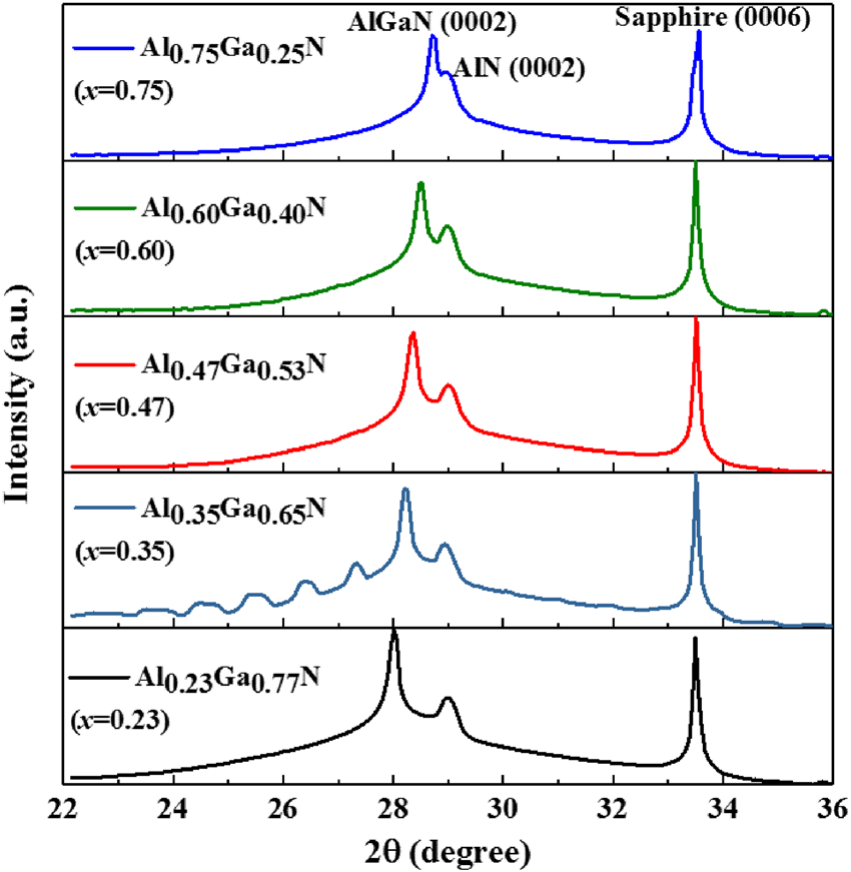
HRXRD 2θ−ω scan near (0002) Bragg reflection plane for the Al_*x*_Ga_1−*x*_N thin films.

The crystal structure and lattice parameters of MOCVD-grown Al_*x*_Ga_1−*x*_N and AlN have been studied using HRXRD and RSM techniques, while photoluminescence and Raman measurement results are discussed to understand the bandgap and phonon modes in Al_*x*_Ga_1−*x*_N and AlN. Figure 1 shows the 2θ-ω Bragg reflections (*λ* = 1.23984 Å) around (0002) crystal planes for Al_*x*_Ga_1−*x*_N with varying *x*. The effect of strain is taken into account to determine the *x* values as per the synchrotron radiation HRXRD results^33^. Bragg reflection peaks of (0002) from Al_*x*_Ga_1−*x*_N and AlN, and of (0006) from the sapphire substrate, are observed. The satellite peaks or the Laue oscillations in Al_0.35_Ga_0.65_N could be due to relatively smoother surface of Al_x_Ga_1−x_N with 35% Al or due to the scattering of x-rays within the Al_0.35_Ga_0.65_N and the AlN layers. However, the primary goal here is to investigate the effect of Al content on the dominant and defining (0002) peak in the epitaxial layers.

The out-of-plane *c*-axis lattice constant (*c*) of Al_x_Ga_1−x_N thin films were calculated as shown in Table 1. Vegard’s law provides reliable unstrained lattice constants (*c*_0_, *a*_0_) for Al_*x*_Ga_1−*x*_N films using the bandgaps of GaN and AlN, and considering the very small lattice mismatch (~2%) between GaN and AlN^19,33–36^. The calculated *c*, is lower than the unstrained *c*_0_, indicating a compressive strain along the *c*-axis (out-of-plane) in the Al_*x*_Ga_1−*x*_N thin films.

**Table 1:** Calculated strained (*a*, *c*) parameters (from HRXRD 2θ−ω scan and asymmetric RSM scans) and unstrained lattice parameters (*a*_0_, *c*_0_) (from Vegard’s law), Al composition (*x*)^33^, elastic constants (*C*_11_, *C*_12_, *C*_13_, and *C*_33_) and Poisson ratio (*υ*) of Al_*x*_Ga_1−*x*_N. (${\nu }_{0}^{AlN}$ = 0.207 and ${\nu }_{0}^{GaN}$ = 0.202)

Al composition (*x*)	In-plane lattice parameter [Å]		Out-of-plane lattice parameter [Å]		Elastic constant [GPa]				Poisson ratio (*υ*)
	Calculated (*a*)	Unstrained (*a*_0_)	Calculated (*c*)	Unstrained (*c*_0_)	*C*_11_	*C*_12_	*C*_13_	*C*_33_	
*x* = 0.23	3.190	3.171	5.121	5.138	394.83	145.92	104.39	395.93	0.203
*x* = 0.47	3.185	3.152	5.061	5.088	399.87	146.88	102.71	393.77	0.204
*x* = 0.75	3.169	3.130	4.998	5.031	405.75	148.00	100.75	391.25	0.205

**Figure 2:**
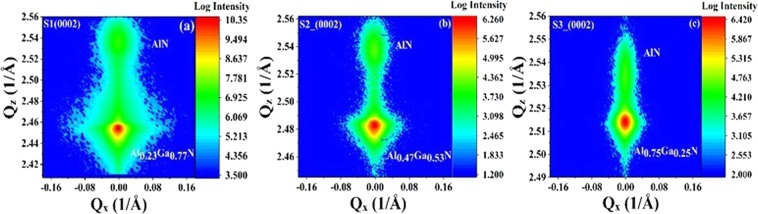
Symmetric RSM (0002) scan of the Al_*x*_Ga_1−*x*_N/AlN thin films.

RSM based analysis were also done to determine the lattice constants and the stress-strain phenomenon in Al_x_Ga_1−x_N with changes in *x*. Figure 2 shows the symmetric plane RSM in the (0002) direction. A clear broadening of Al_*x*_Ga_1−*x*_N reciprocal lattice points (RLPs) reflection intensity distribution towards *Q*_*z*_ and *Q*_*x*_ is seen. It can be observed that the maximum reflection intensity of Al_*x*_Ga_1−*x*_N shifts to higher *Q*_*z*_ values and the lattice constant *c* reduces, as *x* increases, which agrees very well with the results obtained from the 2θ-ω scan. Also, broadening along the Q_z_ direction increases with *x*. Changes in the RSM plots with different Al content seem to be dominated by the Al_*x*_Ga_1−*x*_N layer.

**Figure 3:**
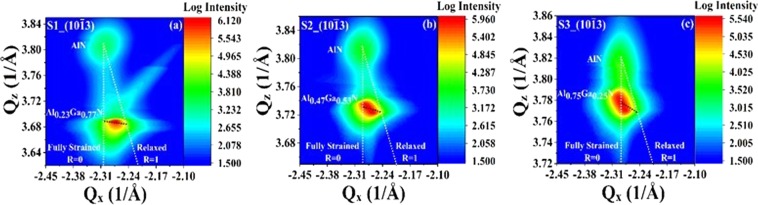
Asymmetric RSM (10$\bar{1}$3) scan of the Al_*x*_Ga_1−*x*_N/AlN thin films grown on sapphire. (**a**) Al_0.23_Ga_0.77_N, (**b**) Al_0.47_Ga_0.53_N, and (**c**) Al_0.75_Ga_0.25_N. The dashed white lines indicate where the fully relaxed (*R* = 1) and fully strained (*R* = 0) Al_*x*_Ga_1−*x*_N layers with varying Al compositions should be. The dashed black lines show the relaxation directions in the reciprocal space for different Al compositions.

Reciprocal space map around the AlN asymmetric (10$\bar{1}$3) RLP is illustrated in Figure 3. Based on the information from the asymmetric RSM scan, lattice parameters (*a* and *c*) were calculated for the hexagonal structure Eq. (1)^37–39^:

1 $$a=\frac{2\pi }{|{Q}_{x}|}\sqrt{\frac{4({h}^{2}+{k}^{2}+hk)}{3}},\,c=\frac{2\pi l}{{Q}_{Z}},$$

Table 1 presents the calculated lattice parameters from the asymmetric RSM measurement (in this particular case, *h* = 1, *k* = 0, and *l* = 3) for Al_*x*_Ga_1−*x*_N. The calculated *c* from asymmetric RSMs is very close to the one obtained by HRXRD 2θ−ω scans for each sample, with a difference of about 0.06%; hence only the c-parameters from the HRXRD results are shown. The calculated *a* is larger than the unstrained one (*a*_0_) obtained by Vegard’s law, which is due to the tensile strain along the *a*-axis (in-plane) in the Al_*x*_Ga_1−*x*_N epitaxial layers. Also, the *a*-lattice constant reduces with an increase in *x*, similar to *c*. A reduction in the lattice size and increase in the strain is seen in Al_*x*_Ga_1−*x*_N with an increase in the Al content in the alloy.

Figure 3 shows that with increasing Al composition, the maximum reflection intensity of Al_*x*_Ga_1−*x*_N RLPs progressively shifts from a partially relaxed (R = 1) towards a fully strained (R = 0) position. Since the AlN layer is thinner (~120 nm) than the Al_x_Ga_1−x_N layer (~800 nm), its reflection peak intensity is lower than Al_*x*_Ga_1−*x*_N. The intensity of Al_x_Ga_1−x_N RLP broadens along the direction associated with the relaxation of the layer (the dashed black line). The Al_*x*_Ga_1−*x*_N RLPs get closer to the fully strained position with an increase in *x*. Note that both AlN and Al_0.75_Ga_0.25_N have a similar *Q*_*x*_ value of −2.38 Å^−1^. An increase in the strain is observed with Al incorporation in Al_*x*_Ga_1−*x*_N, despite of reductions in lattice mismatch. As seen in Figure 3, a strain complementary to Al_*x*_Ga_1−*x*_N is induced in the AlN intermediate layer which increases with *x* as the Al_x_Ga_1−x_N layer is relaxed and adds to the inherent strain that is already present in AlN. The broadening in the symmetric and asymmetric RLPs implies an increase in the screw and edge dislocations (which are in the order of 10^8^–10^9^ cm^−2^) respectively with *x*. The RSM and the 2θ-ω results show that the dislocations and the coherence lengths in Al_x_Ga_1−x_N/AlN change with *x*. Lattice constants of hexagonal AlN are typically smaller than GaN and hence, a reduction in the lateral correlation lengths and an increase in the dislocations are seen as the Al_*x*_Ga_1−*x*_N composition is varied from GaN to AlN.

The overall in-plane strain (*ε*_*a*_) and out-of-plane strain (*ε*_*c*_) in the Al_*x*_Ga_1−*x*_N layers were determined using Eq. (2)^38,40–42^:

2 $${\varepsilon }_{a}=\frac{a-{a}_{0}}{{a}_{0}},\,{\varepsilon }_{c}=\frac{c-{c}_{0}}{{c}_{0}},$$

The calculated strains (*ε*_*a*_ and *ε*_*c*_) are attributed to the biaxial (${\varepsilon }_{a}^{b}$ and ${\varepsilon }_{c}^{b}$) and hydrostatic (*ε*_*h*_) strains as shown in Eq. (3)^34,36^. (${\varepsilon }_{a}^{b}$ and ${\varepsilon }_{c}^{b}$ are the biaxial strains along *a*- and *c*-directions, respectively.)

3 $${\varepsilon }_{a}={\varepsilon }_{a}^{b}+{\varepsilon }_{h},\,{\varepsilon }_{c}={\varepsilon }_{c}^{b}+{\varepsilon }_{h},$$

where *ε*_*h*_ is defined as ${\varepsilon }_{h}=\frac{1-\upsilon }{1+\upsilon }({\varepsilon }_{c}+\frac{2\upsilon }{1-\upsilon })$, $\upsilon$ is Poisson’s ratio of Al_*x*_Ga_1−*x*_N calculated using Vegard’s law $({v}_{AlGaN}(x)={x}_{AlN}+{(1-x)}_{GaN})$^43^ and shown in Table 1. For the hexagonal crystal structure, the in-plane biaxial stress (*σ*^*b*^) in the Al_x_Ga_1−x_N epitaxial layer can be determined by *σ*^*b*^ = *M*_*b*_${\varepsilon }_{a}^{b}$, where *M*_*b*_ is the biaxial elastic modulus given by ${M}_{b}=({C}_{11}+{C}_{12}+2\frac{{C}_{13}^{2}}{{C}_{33}})$^41^. The elastic constants (*C*_*ij*_) of Al_*x*_Ga_1−*x*_N (Table 1) can be obtained by Vegard’s law (${C}_{ij}^{AlGaN}(x)=x{C}_{ij}^{AlN}+(1-x){C}_{ij}^{GaN})\,$^44,45^. The calculated strains, biaxial strains, hydrostatic strain, and biaxial stress for Al_x_Ga_1−x_N epitaxial layers are summarized in Table 2. It can be seen that the in-plane (biaxial) strains are tensile, while the out-of-plane (biaxial) strains are compressive because of the different lattice mismatch along the in-plane and out-of-plane axes^19^ as also seen in the HRXRD results.

**Table 2:** Measured in-plane and out-of-plane strains, biaxial strains, hydrostatic strain, and biaxial stress of Al_*x*_Ga_1−*x*_N. Positive and negative values denote tensile and compressive strains respectively.

Al composition (*x*)	In-plane strain (*ε*_*a*_) [%]	In-plane biaxial strain (${\varepsilon }_{a}^{b}$) [%]	Out-of-plane strain (*ε*_*c*_) [%]	Out-of-plane biaxial strain (${\varepsilon }_{c}^{b}$) [%]	Hydrostatic strain (*ε*_*h*_)	Biaxial stress (*σ*^*b*^) [GPa]
*x* = 0.23	0.6	0.6	−0.3	−0.3	1.06 × 10^−6^	2.9
*x* = 0.47	1.0	1.0	−0.5	−0.5	−2.35 × 10^−5^	5.1
*x* = 0.75	1.2	1.2	−0.6	−0.6	−3.50 × 10^−6^	6.3

The biaxial strain has values close to the total strain in Al_*x*_Ga_1−*x*_N due to the relatively smaller values of *ε*_*h*_ and very few impurities introduced during growth. Also, the full width at half maximum (FWHM) values of the HRXRD (0002) ω scans (not shown here) are found to be 627, 642, and 847 arcsec for Al_0.23_Ga_0.77_N, Al_0.47_Ga_0.53_N, and Al_0.75_Ga_0.25_N, respectively (Table 3)^32^. The lateral coherence lengths would range from 100 nm to 200 nm and have inverse proportionality with the Al content, indicating that the Al_*x*_Ga_1−*x*_N samples used in this study are of good crystal quality.

**Table 3:** Summary of structural and optical results of the Al_*x*_Ga_1−*x*_N thin films.

Al composition (*x*)	FWHM of HRXRD [arcsec]	Screw TD Density [cm^−2^]	FWHM of PL [meV]	Energy gap [eV]
*x* = 0.23	627	7.9 × 10^8^	74	3.88
*x* = 0.47	642	8.3 × 10^8^	100	4.27
*x* = 0.75	847	1.4 × 10^9^	206	5.25

**Figure 4:**
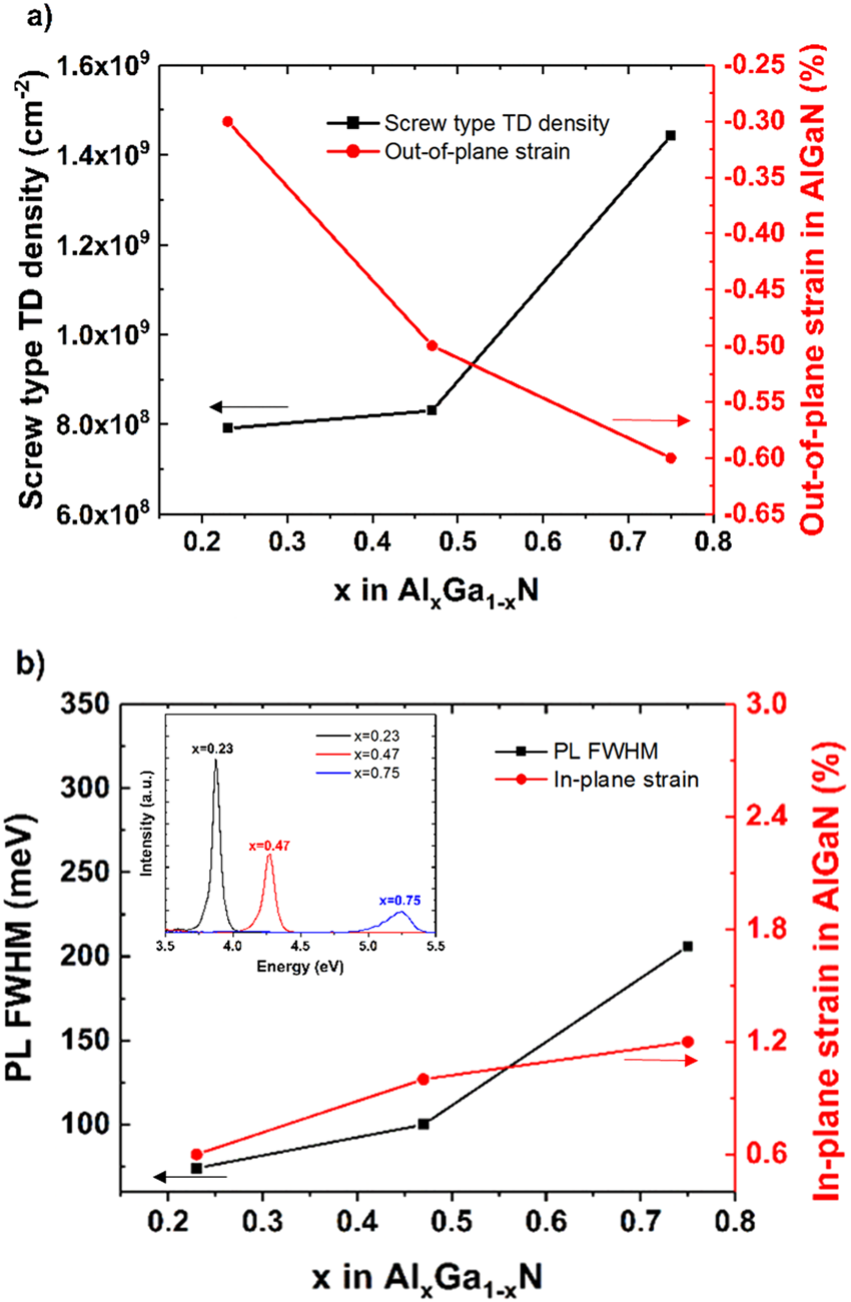
Compositional dependence of (**a**) screw (*c*-type) TD density and out-of-plane strain, (**b**) PL FWHM and in-plane strain of Al_*x*_Ga_1−*x*_N layers. The inset shows the room temperature PL spectra of Al_0.23_Ga_0.77_N, Al_0.47_Ga_0.53_N, and Al_0.75_Ga_0.25_N.

The broadening of the FWHM of (0002) HRXRD ω scans in Al_*x*_Ga_1−*x*_N could be associated with the screw (*c*-type) threading dislocation (TD) along the *c*-axis. Figure 4(a) presents the compositional dependence of screw (*c*-type) TD density and out-of-plane strain in the Al_*x*_Ga_1−*x*_N thin films. The dislocation density of the Al_*x*_Ga_1−*x*_N thin films can be estimated from:

4 $${D}_{screw}=\frac{{\beta }_{(0002)}^{2}}{4.35{b}_{screw}^{2}},$$

where *D*_*screw*_ is the screw type TD^24^, *β* is the FWHM of the (0002) ω scan, and *b*_*screw*_ = 5.1855 Å is the Burgers vector length for screw-type TD. As *x* increases, both the screw type TD density and the strain increase (Fig. 4(a)). Evidently, the high density of screw dislocation observed in the Al-rich samples originated from a compressive strain along the *c*-axis (up to 0.6%) and a biaxial stress (up to 6.313 GPa), in Al_x_Ga_1−x_N, as presented in Table 3.

Photoluminescence measurements (Figure 4(b)) further indicate and help to understand the strain and stress in the epitaxial layers. A broadening of the Al_x_Ga_1−x_N peaks in observed with an increase in *x*. Also, there is a shift in the peak positions compared to the unstrained energy gaps that are predicted by Vegard’s law. The PL peak positions are measured at 3.88, 4.27, and 5.25 eV for Al_0.23_Ga_0.77_N, Al_0.47_Ga_0.53_N, and Al_0.75_Ga_0.25_N, respectively. According to Vegard’s law, the predicted energy gap values for *x* = 0.23, 0.47, and 0.75 are 4.06, 4.73, and 5.51 eV respectively (considering E_g_(AlN) = 6.2 eV, E_g_(GaN) = 3.42 eV). If a bowing parameter of 1 eV is taken into consideration^46^, the predicted bandgap values are 3.88, 4.47, and 5.32 eV for *x* = 0.23, 0.47, and 0.75, respectively. Smaller bandgap in the measured samples as compared to the predicted values, could be attributed more to the stronger tensile strain effect along the *a*-axis direction than the *c*-axis compressive strain (*ε*_*a*_ ≈ 2*ε*_*c*_) in the Al_*x*_Ga_1−*x*_N epitaxial layers and hence, to the overall larger lattice constants of Al_x_Ga_1−x_N epitaxial layers as compared to unstrained Al_*x*_Ga_1−*x*_N. The difference between the predicted and measured bandgap values is more for *x* = 0.47 and 0.75 than *x* = 0.23 due to more residual strain in Al_x_Ga_1−x_N with high Al composition. Also, the bandgap increases with *x* as would be expected and seems to be tunable between GaN and AlN. The PL peak broadening, intensity suppression and peak shifts could have multiple origins such as a statistical variation in the composition, Al-induced alloy disorder, strain and dislocations.

**Figure 5:**
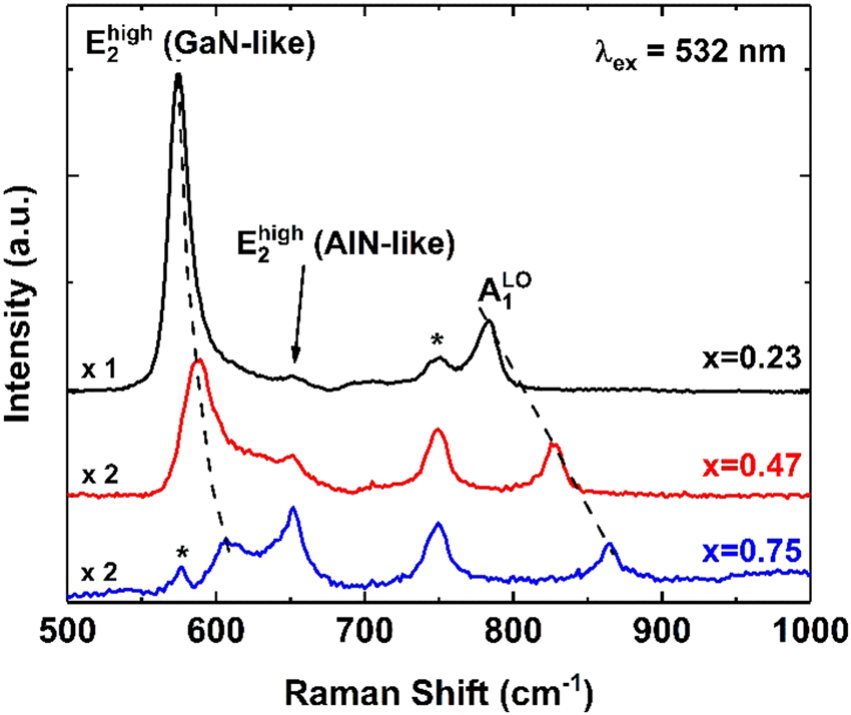
Raman spectra for Al_*x*_Ga_1−*x*_N/AlN thin films (*x* = 0.23, 0.47, 0.75) measured with a 532 nm excitation laser under ambient conditions. The Raman spectra for *x* = 0.47 and 0.75 are multiplied by a factor of two for clarity. The dashed lines marking the composition dependence of the E_2_^high^ (GaN-like) and A_1_^LO^ modes are guides to the eye. Asterisks near 576 (only observable for *x* = 0.75 because of overlapping with the E_2_^high^ (GaN-like) mode) and 750 cm^−1^ show the *c*-plane sapphire substrate phonons.

Raman spectra of the Al_*x*_Ga_1−*x*_N samples under 532 nm excitation are shown in Figure 5. Two-mode behavior for the E_2_^high^ phonon^47^ and one-mode behavior for the A_1_^LO^ phonon^48^ are seen. Here, E_2_^high^ and A_1_^LO^ phonon modes correspond to the atomic oscillations in the *c*-plane (parallel to the *a*-axis) and along the *c*-axis, respectively. The phonon peaks exhibit a shift with increasing *x*. The E_2_^high^ (GaN-like) phonon is located at 575, 587, and 607 cm^−1^ for *x* = 0.23, 0.47, and 0.75, respectively, while the E_2_^high^ (AlN-like) phonon is located at ∼650 cm^−1^ with a weak composition dependence. The A_1_^LO^ phonon also exhibits strong composition dependence, from 783 to 864 cm^−1^ when *x* increases from 0.23 to 0.75. A sharp peak at 750 cm^−1^ (marked with an asterisk) and a weak peak at 576 cm^−1^ (marked with an asterisk and most visible for *x* = 0.75 because the peak is overlaid by the strong E_2_^high^ (GaN-like) peak) correspond to phonon vibrations of the the sapphire substrate. The composition-dependence behavior of the E_2_^high^ (GaN-like) and A_1_^LO^ modes is in good agreement with previous work on Al_*x*_Ga_1−*x*_N epitaxial layers^48–50^wherein the Raman results also confirm the wurtzite structure of the Al_*x*_Ga_1−*x*_N layer with its hexagonal [0001] crystal plane parallel to the *c*-plane sapphire substrate. Strain due to alloying seems to be the major mechanism for the observed Raman shifts (the difference in phonon energies due to substrate-induced strain is small). Moreover, the E_2_^high^ (AlN-like) peak intensity varies with *x*, as the phonon vibrations are sensitive to atom compositions. Therefore, higher *x* values revealed more distinct E_2_^high^ (AlN-like) phonon vibration peaks, which is typical of alloy semiconductors. The result also suggests that the AlN buffer layer quality is good, so there is a small substrate-induced strain in the Al_*x*_Ga_1−*x*_N epitaxial layers.

## Conclusion

In summary, the study focuses on the strain-stress status of Al_*x*_Ga_1−*x*_N epitaxial layer grown by MOCVD on a *c*-plane sapphire substrate with AlN as intermediate layers. The lattice parameters reduce as the Al content in Al_*x*_Ga_1−*x*_N is increased. The out-of-plane strain of Al_*x*_Ga_1−*x*_N is compressive, and the in-plane strain is tensile. The strain increases with *x*, even though the lattice mismatch between Al_*x*_Ga_1−*x*_N and AlN reduces. Broadening of the RSM peaks and the HRXRD rocking curve scans imply a consistent reduction in correlation lengths and higher dislocation densities with increasing *x* as the Al_*x*_Ga_1−*x*_N composition is varied from GaN to AlN. The bandgap of Al_x_Ga_1−x_N increases with *x*, as expected. Also, the values are smaller than the unstrained bandgap predicted by Vegard’s law, due to a larger tensile strain on the *a*-axis compared to the compressive strain on the *c*-axis. The E_2_^high^ and LO phonons exhibit a shift with an increasing *x* caused due to the strain accompanied with alloying. Considering the potential of Al_*x*_Ga_1−*x*_N for optical and electronic applications, this work adds towards the understanding of crystal and optical properties of Al_x_Ga_1−x1−x_N/AlN structure with high *x*; which need to be addressed or utilized for the development of optimum Al_*x*_Ga_1−*x*_N/AlN based devices.

## Methods

### Metal-organic chemical vapor deposition (MOCVD) growth

Al_*x*_Ga_1−*x*_N thin films with varying *x* were grown on *c*-plane sapphire substrates by metal-organic chemical vapor deposition (MOCVD). The precursors for Al, Ga, and N, are trimethylaluminum (TMA), trimethylgallium (TMG), and ammonia (NH_3_), respectively. To remove surface contamination, sapphire substrates were heated at 1100 °C in H_2_ ambient prior to the growth. A 40 Torr chamber pressure was maintained for the growth of AlN and Al_*x*_Ga_1−*x*_N epitaxial layers. A ~20 nm low-temperature (LT) AlN nucleation layer with a V/III ratio of 3000 was deposited on the sapphire substrate at 600 °C. The temperature was then increased to 1040 °C to grow a ~100 nm high-temperature (HT) AlN buffer layer. Finally, a ~800 nm Al_*x*_Ga_1−*x*_N epitaxial layer was grown on the AlN layer at 1140 °C^3^. The samples were cooled in NH_3_ environment.

### Materials characterizations

Synchrotron radiation HRXRD measurement were performed at 33IDD beamline at the Advanced Photon Source, Argonne National Laboratory. It is equipped with a standard six-circle Kappa-type diffractometer and Pilatus 100 K area detector. A deep ultraviolet (DUV) PL spectroscopy (excitation at 224 nm) was used to measure the optical properties of the Al_x_Ga_1−x_N thin films. Micro-Raman spectroscopy was performed using a Horiba Jobin-Yvon Xplora confocal Raman spectrometer in a backscattering configuration with a 532 nm excitation laser and a grating of 1800 lines/mm.

## Data Availability

The datasets generated during and/or analyzed in the current study are available from the corresponding author on reasonable request.
